# Durable Complete Response to Pembrolizumab in Esophageal Squamous Cell Carcinoma With Divergent Microsatellite Status: A Case Report

**DOI:** 10.3389/fonc.2021.767957

**Published:** 2021-11-19

**Authors:** Tian Zeng, Lei Zhang, Can Chen, Xiang Zhao, Xiaoqing Liu, Fengwei Ran, Tingting Yong, Ying Yang, Henghui Zhang, Yanling Zhang

**Affiliations:** ^1^ Department of Oncology, Southwest Hospital, Third Military Medical University (Army Medical University), Chongqing, China; ^2^ Genecast Biotechnology Co., Ltd., Wuxi, China; ^3^ Beijing Shijitan Hospital, Capital Medical University, Beijing, China; ^4^ Ninth School of Clinical Medicine, Peking University, Beijing, China; ^5^ School of Oncology, Capital Medical University, Beijing, China

**Keywords:** esophageal squamous cell carcinoma, microsatellite instability, pembrolizumab, complete response, DNA damage repair

## Abstract

Microsatellite instability-high (MSI-H) is widely believed to be a biomarker for immune checkpoint inhibitors (ICIs) such as pembrolizumab in solid tumors. However, due to the low prevalence of MSI-H in most cancers, it tends to be insufficient to identify whether patients should receive ICIs according to this biomarker alone. Here, we report a Chinese esophageal squamous cell carcinoma (ESCC) patient with unusual divergent MSI status between the primary lesion (MSS) and metastatic lesion (MSI-H) which developed after platinum-based therapy and radiotherapy. Both his primary and metastatic tumors responded well to pembrolizumab-containing therapies or pembrolizumab monotherapy and maintained a complete response for over 24 months. Whole-exome sequencing and multiplex immunohistochemistry were used to examine his tissue specimens. Notably, there were multiple high-frequency mutations of DDR (DNA damage repair) genes shared in the primary lesion and metastatic lesion, especially in the latter. Besides, we observed considerable degrees of infiltrating CD3^+^/CD8^+^ lymphocytes in both of his primary tumor and metastatic tumor without obvious difference, suggesting that the conversion of microsatellite status had little effect on the infiltration of lymphocytes. Collectively, given the predictive role of DDR alterations for ICIs in other malignancies, the alterations of DDR genes might also be promising biomarkers in ESCC individuals receiving ICIs.

## Highlights

Mutations of DDR (DNA damage repair) genes may be biomarkers for checkpoint blockade immunotherapy in esophageal squamous cell carcinoma (ESCC).

## Introduction

Prior studies (1, 2) demonstrated improved responses to immune checkpoint blockade (ICB) in multiple solid tumors with mismatch-repair deficiency (dMMR) or microsatellite instability-high (MSI-H). Hence, pembrolizumab monotherapy has been approved by the FDA for dMMR or MSI-H patients with solid tumors, irrespective of the tissue of origin since 2017. However, the prevalence of MSI-H/dMMR is rather low in esophageal and esophagogastric junction carcinoma (2), implying that this biomarker alone may be insufficient to identify appropriate patients with esophageal cancer who could receive immune checkpoint inhibitors (ICIs). Herein, we encountered a Chinese esophageal squamous cell carcinoma (ESCC) patient who had unusual divergent MSI status between the primary lesion (MSS) and metastatic lesion (MSI-H). Surprisingly, he exhibited durable complete response (>24 months) to pembrolizumab in the setting of neck metastasis which developed after receiving platinum-based therapy and radiotherapy. To explore what accounted for this patient outcome, whole-exome sequencing (WES) and multiplex immunohistochemistry (mIHC) were used to characterize his genomic features and immune phenotypes in the tumor microenvironment.

## Case Presentation

**Figure 1:**
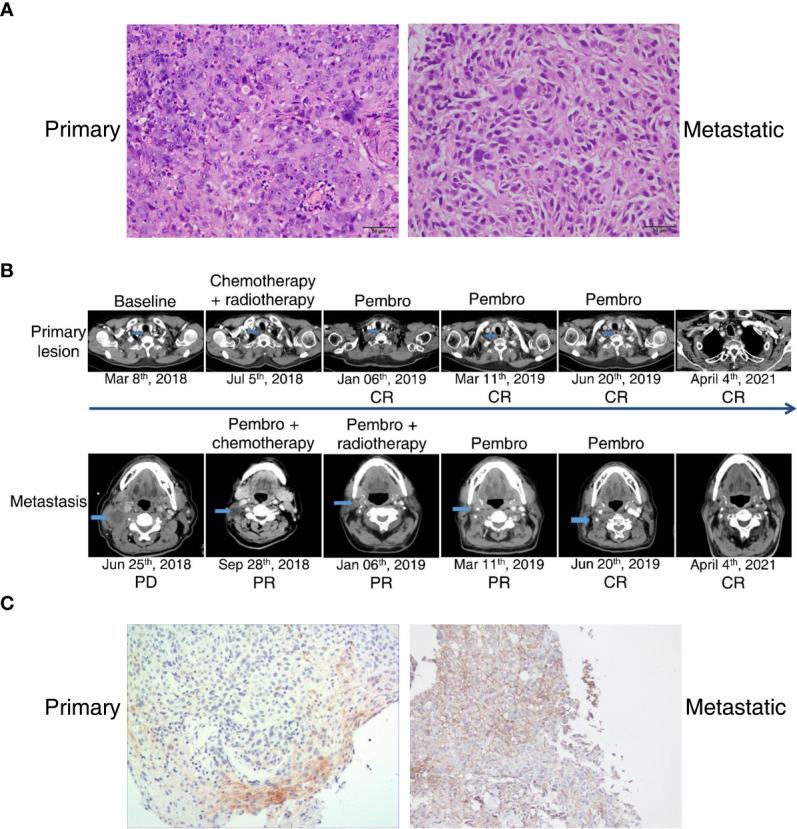
Pathological results, radiological images, and PD-L1 assay of an esophageal squamous carcinoma patient with divergent MSI status between primary and metastatic lesions. **(A)** Histology of his primary and metastatic tumors. Magnification, ×200. **(B)** Representative CT images associated with his clinical outcomes during the whole treatment. Outcomes were valuated according to RECIST 1.1 criteria. **(C)** IHC staining with anti-PD-L1 antibody (SP142). Magnification, ×100.

**Figure 2:**
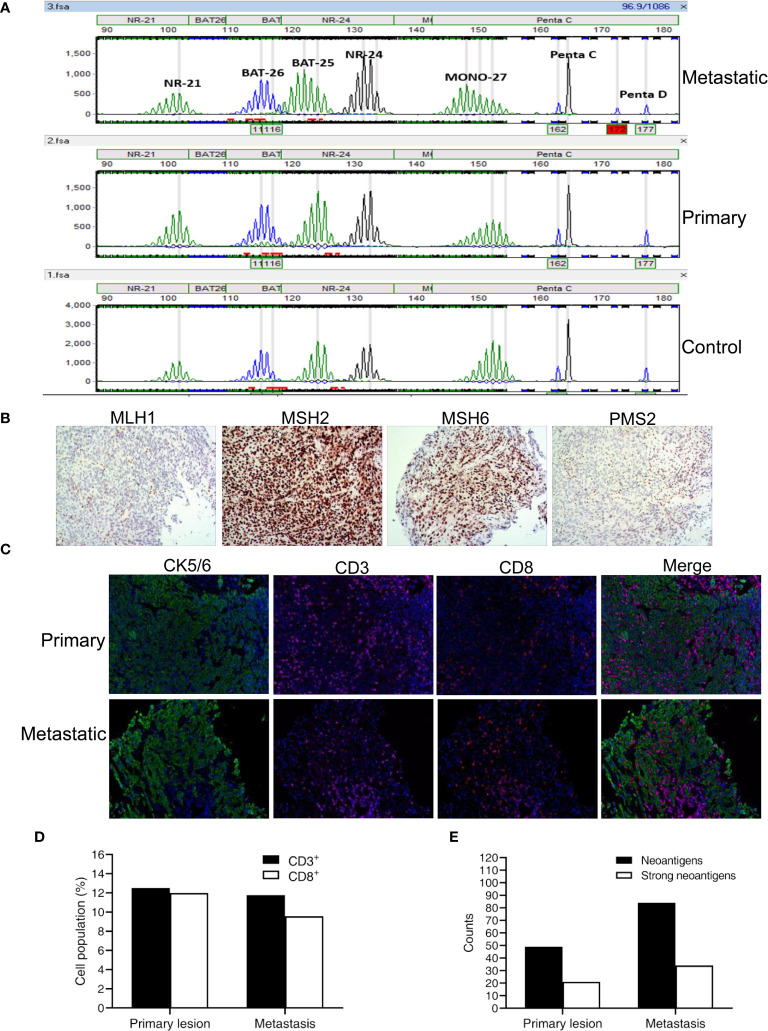
MSI status, MMR results, and immune phenotypes together with neoantigens in primary and metastatic lesions of this patient. **(A)** Detection results of five microsatellite sites by the MSI analysis system (Promega). **(B)** MMR results of MLH1, MSH2, MSH6, and PMS2 in metastasis. **(C)** Representative images of CK5/6 (cytokeratin 5/6), CD3, and CD8 shown by mIHC in primary and metastatic lesions. Nuclei (blue) were counterstained by DAPI. Magnification, ×200. **(D)** Quantification results of mIHC from 15 random vision fields. **(E)** The number of predicted neoantigens in primary and metastatic tumors.

In February 2018, a 73-year-old Chinese man presented with a rough mass in his esophageal mucosa and surface hyperemia under gastroscopy. A biopsy sample of pathological diagnosis showed ESCC (**Figure 1A**). A computed tomography (CT) scan revealed obvious contrast-enhanced imaging of the upper esophagus with a thickening wall and narrowing lumen but without visible enlargement of the mediastinal lymph nodes (**Figure 1B**), and he was diagnosed with a T2N0M0, upper esophageal carcinoma. His biopsy tumor specimens were sent for next-generation sequencing (NGS) targeting 543 cancer-related genes as well as a PD-L1 (programmed death-ligand 1) assay at a CAP (College of American Pathologists)-certified laboratory (Genecast Biotechnology Co., Ltd.). Unfortunately, no actionable alterations with strong evidence for targeted therapy were identified in his primary lesion, where the expression level of PD-L1 (SP142 staining) was 5% (CPS close to 6) and microsatellite stability (MSS) was present (**Figures 1C**, **2A**). Owing to the upper location of his tumor, this patient was unable to receive surgery. Given the fact that he had radioactive esophagitis and tracheitis (grade 3) during radiotherapy, we adjusted the originally planned therapeutic regimen, and sequential chemotherapy plus radiotherapy rather than concomitant definitive chemoradiation was used for him. Specifically, he received 3 cycles of paclitaxel liposome plus nedaplatin for 2 months followed by radical intensity-modulated radiotherapy (IMRT) (66 Gy at 2.2 Gy/session to the esophageal tumor) for 1 month until early July 2018 (**Figure 1B**). Nevertheless, the CT scan showed multiple enlarged lymph nodes of the right-side neck with suspected metastasis on June 25, 2018 (**Figure 1B**), which was verified by pathological diagnosis of a puncture biopsy of the right cervical mass (**Figure 1A**). Next, his punctured samples of the metastatic tumor were sent to the same laboratory as before for another NGS test and PD-L1 assay. Surprisingly, MSI-H along with dMMR (**Figures 2A, B**) was observed in his metastatic tumor with the soaring percentage (TPS 70%, CPS 75) of PD-L1 (**Figure 1C**). According to these results, the patient was given adjusted treatments with pembrolizumab (100 mg, recommended dose 2 mg/kg at that time in China) combined with chemotherapy (docetaxel plus tegafur) or local IMRT (66 Gy at 2.2 Gy/session to the metastatic tumor) or single pembrolizumab treatment from July 2018 to March 2019 and exhibited sustained clinical response, regardless of primary or metastatic lesions (**Figure 1B**). Given the good response after adding pembrolizumab, this patient did not continue to undergo chemotherapy or radiotherapy but just maintained pembrolizumab (100 mg) monotherapy until June 2019 and achieved a complete response (CR) of both primary and metastatic tumors (**Figure 1B**). Subsequently, he stopped the above therapies in June 2019 and remained CR until the recent radiological evaluation in April 2021 (**Figure 1B**).

## Methods

WES and variant analysis of the carcinoma tissue specimens of the patient along with a peripheral blood sample were performed at a CAP-certified laboratory (Genecast Biotechnology Co., Ltd.). The tumor cellular contents of both primary and metastatic biopsies were >80%. DNA libraries were captured by a Roche NimbleGen SepCap EZ MedExome kit and sequenced on a NovaSeq 6000 system (Illumina) with a deduped depth of 200× in tissues. Somatic SNV calling was performed using GATK (v4.0.7.0, Mutect2) (3) and the alterations were annotated by Oncotator (4) and ANNOVAR (5). For neoantigen prediction, *HLA*-I (*A*, *B*, and *C*) genotyping based on WES data of genomic DNA of peripheral blood cells was performed using the HLA-HD algorithm (6). Novel 9–11 amino-acid peptides from somatic non-synonymous mutations detected in tumor tissues were determined, and binding affinities of mutant or wild-type peptides to *HLA*-I alleles were calculated *via* netMHC-4.0 (7). The novel peptides with predicted binding affinities <500 or 50 nm and the affinity ratio of wild-type peptides to mutant peptides >1.5 were defined as neoantigens or strong neoantigens (8), respectively. For the examination of the tumor immune microenvironment by mIHC, immunostaining, imaging, and quantitative analyses of multiple molecules in formalin-fixed paraffin-embedded (FFPE) tumor slides were performed as previously described (9, 10). Primary antibodies used in this work were listed as follows (clone name, catalog number, dilution ratio): CK5/6 (OTI1C7, ZM-0313, 1:100), CD3 (LN10, ZM-0417, 1:100), and CD8 (SP16, ZA0508, 1:100). All of them were purchased from ZSGB-BIO, Beijing, China. The stained slides were scanned by PerkinElmer Vectra (Vectra 3.0.5; PerkinElmer, MA, USA) and quantitative results were achieved by inForm Advanced Image Analysis software (inForm 2.3.0; PerkinElmer, MA, USA). For each slide, more than 15 fields were included to count the number, percentage, and density of positive cells in all nucleated cells under ×200 magnification. Mean percentages of certain cell subsets were calculated for further analysis.

## Results and Discussion

The discrepancy of microsatellite status detected by the MSI analysis system (Promega) between the primary and metastatic tumors of the patient was observed by targeted multigene panel sequencing as well. Based on calculation by the MSI-sensor software with WES data, the MSI scores of the primary and metastatic tumors were 2.22 and 21.11, respectively. According to the cutoff value (>10) for judging MS instability, the MS status of the primary tumor was judged as MSS, while the MS status of the metastatic tumor was judged as MSI-H. Besides, the TMB (tumor mutation burden) value was higher in the metastatic (18.25/Mb) than in the primary tumor (9.69/Mb) and it is consistent with their divergent MS status. Microsatellite instability is universally believed as the consequence of deficiency of four major MMR proteins encoded by *MLH1*, *MSH2*, *MSH6*, and *PMS2* (11), whereas there were not any detrimental alterations of these four genes identified in the above two NGS tests. To further examine the potential causes for this divergent MSI status, we performed WES of the tissue samples of the patient. As expected, WES results also displayed that MSI-H and MSS were present in metastatic and primary tumors, respectively. Notably, this individual had a lot of somatic alterations in well-documented DDR (DNA damage repair) genes (12) whose mutation frequencies were generally much higher in the metastatic than in the primary lesion, and three mutated DDR genes (*RAD51C*, *RECQL4*, *RMI1*) were only present in the former but absent in the latter (**Table 1**). All the variants were screened in public databases (ClinVar, OncoKB, COSMIC, CIViC), and almost all of them had uncertain significance except *TP53* p.R282W which was classified as pathogenic mutation. In view of the crucial role of DDR genes including MMR-related genes in maintaining genome integrity (12), we could speculate that chemotherapy as well as radiotherapy before immunotherapy might fuel the generation of high-frequency DDR alterations, further aggravating genome instability in the metastatic tumor relative to the primary tumor. Accordingly, microsatellite status was reshaped from MSS to MSI-H, though the detailed mechanisms remain to be studied.

**Table 1:** Alterations of DDR (DNA damage repair) genes in primary and metastatic lesions.

Alterations of DDR genes	Primary lesion (Fre, %)	Metastasis lesion (Fre, %)	DDR pathway	Core DDR pathway
***ERCC2* p.R666W**	10.88	28.1	NER	NER
***FANCM* p.Q740L**	7.62	28.28	FA, HR	FA
***RIF1* p.R2202C**	13.48	30.06	NHEJ, other	
***TP53* p.R282W**	28.09	77.59	Other	
*TTK* p.R854Gfs*39	11.11	25.49	Other	
***PRKDC* p.M2429I**	5.39	21.8	NHEJ	NHEJ
***PRKDC* p.M2428I**	5.63	22.83	NHEJ	NHEJ
*PRKDC* p.L934Ffs*46	10.87	22.88	NHEJ	NHEJ
*PRKDC* p.D2431Tfs*10		20.14	NHEJ	NHEJ
***RAD51C* p.L265P**		6.31	FA, HR	
***RECQL4* c.1132-1G>T**		5.75	HR	
*RMI1* p.N377Mfs*6		7	FA, HR	

Fre, frequency; NER, nucleotide excision repair; FA, Fanconi anemia; HR, homology-dependent recombination; NHEJ, non-homologous end joining.

Despite the lasting clinical response of this individual since adding pembrolizumab, can it be fully attributed to MSI-H status in metastasis? If so, it appeared to be unable to account for why the primary tumor with MSS of this patient likewise responded well. Theoretically, in addition to PD-L1, tumor-infiltrating lymphocytes (TILs) are also indispensable to successful ICB-based therapies (13). Indeed, as shown by mIHC images, there were considerable degrees of infiltrating CD3^+^/CD8^+^ lymphocytes in both the primary lesion and metastatic lesion (**Figure 2C**). Besides, quantitative results revealed that the percentages of CD3^+^/CD8^+^ TILs were even slightly higher in the former than in the latter (**Figure 2D**). In other words, the conversion of microsatellite status seemed to have little effect on the profiles of TILs. Therefore, it is reasonable to infer that MSI-H is not mainly responsible for such responsiveness to immunotherapy alone or combined with chemotherapy or radiotherapy, at least not the sole determinant. Furthermore, after discontinuation of all prior therapies since June 2019, this patient still achieved complete remission lasting over 24 months and survival of 40 months until now. This exceptional outcome was obviously better than that (median overall survival of 2.3 years) observed in ESCC patients with definitive chemoradiotherapy (14), indicating the potential of ICIs in improving the survival of ESCC patients. In the context of MSS primary tumor and MSI-H metastasis, complete response to pembrolizumab could be due to antigen spreading that was reported to be responsible for T-cell responses after initial activation (15). However, whether it is applicable to this patient outcome remains to be further studied. On the other hand, we believed that such good outcomes may be more likely because of a series of shared clonal neoantigens between primary and metastatic sites. Given their similar immune microenvironment is appropriate for immunotherapy, it is hypothesized that if this patient received pembrolizumab from the beginning without visible metastasis, his primary tumor may also have responded well. Although chemotherapy and radiotherapy were in favor of facilitating the release of neoantigens triggering immune response to some extent, pre-existing adaptive resistance mediated by the PD-1/PD-L1 pathway in the primary tumor was also enhanced in metastasis with the PD-L1 expression soaring from 5% to 70%. Thereafter, the anti-PD-1 inhibitor pembrolizumab relieved the restrained activity of CD8 T cells and brought about a durable survival benefit for this patient. In brief, favorable infiltration of T cells potentially associated with certain shared clonal mutations rather than just MS status played a crucial role in these satisfactory immunotherapy outcomes.

It is worth mentioning that alterations of DDR genes are viewed as promising biomarkers for the clinical benefit from ICIs in certain malignancies including lung cancer and urothelial cancer (16, 17), while the predictive value of DDR alterations for ICB treatments in esophageal carcinoma is rarely reported to date. Additionally, we noticed the higher number of predicted neoantigens based on WES data in the metastatic lesion over the primary lesion of the patient (**Figure 2E**), corresponding to the change of MSI status. Notwithstanding, more neoantigens *in silico* has not yet given rise to more TILs in the metastatic tumor compared with the primary tumor. It should be noted that there was a marked rising level of PD-L1 in the metastatic tumor before immunotherapy. In consideration of the favorable infiltration of CD3^+^/CD8^+^ T cells in the tumor microenvironment of the patient, it is plausible that chemotherapy and/or radiotherapy together with DDR alterations not only contributed to keeping the immune phenotype active to some extent but also intensified pre-existing adaptive resistance mediated by the PD-1/PD-L1 pathway (13). Such enhanced expression of PD-L1 was likely associated with the resistance of the metastatic lesion to chemotherapy followed by radiotherapy in the setting of partial response of the primary tumor. Under this circumstance, PD-1 blocking by pembrolizumab subsequently brought about desirable remission of both primary and metastatic lesions. In summary, the alterations of DDR genes may be valuable biomarkers in ESCC patients receiving ICIs Although pembrolizumab has been approved by the FDA for advanced or metastatic ESCC patients with PD-L1 (CPS ≥ 10), it seemed to be incapable of fully explaining the outstanding survival benefit of this individual. Whether anti-PD-1/PD-L1 inhibitors combined with chemotherapy or radiotherapy can achieve better clinical outcomes in those PD-L1-positive ESCC patients harboring DDR alterations deserves more research.

## Data Availability Statement

The WES data presented in this study can be found in online repository. The name of the repository and accession number can be found below: Genome Sequence Archive in National Genomics Data Center and accession number HRA001554 that are publicly accessible at https://bigd.big.ac.cn/gsa-human/browse/HRA001554. Other related data were shown in manuscript or can be obtained from the corresponding author upon reasonable request.

## Ethics Statement

Ethical review and approval was not required for the study on human participants in accordance with the local legislation and institutional requirements. The patients/participants provided their written informed consent to participate in this study. Written informed consent was obtained from the individual(s) for the publication of any potentially identifiable images or data included in this article.

## Author Contributions

Conception and design: TZ, LZ, and YZ. Data interpretation: LZ, YY, and TZ. Bioinformatic analysis: YY. Writing and editing of the manuscript: LZ, TZ, HZ, and YZ. TZ, CC, XZ, XL, FR, and TY identified and treated the patient and collected and presented related clinical data. All authors contributed to the article and approved the submitted version.

## Funding

This study was supported by a grant from the National Key Sci-Tech Special Project of China (No. 2018ZX10302207).

## Conflict of Interest

The authors LZ and YY were employed by the company Genecast Biotechnology Co., Ltd.

The remaining authors declare that the research was conducted in the absence of any commercial or financial relationships that could be construed as a potential conflict of interest.

## Publisher’s Note

All claims expressed in this article are solely those of the authors and do not necessarily represent those of their affiliated organizations, or those of the publisher, the editors and the reviewers. Any product that may be evaluated in this article, or claim that may be made by its manufacturer, is not guaranteed or endorsed by the publisher.

## References

[1] Le DT Uram JN Wang H Bartlett BR Kemberling H Eyring AD. PD-1 Blockade in Tumors With Mismatch-Repair Deficiency. N Engl J Med (2015) 372:2509–20. doi: 10.1056/NEJMoa1500596 PMC4481136 26028255

[2] Le DT Durham JN Smith KN Wang H Bartlett BR Aulakh LK. Mismatch Repair Deficiency Predicts Response of Solid Tumors to PD-1 Blockade. Science (2017) 357:409–13. doi: 10.1126/science.aan6733 PMC5576142 28596308

[3] Cibulskis K Lawrence MS Carter SL Sivachenko A Jaffe D Sougnez C. Sensitive Detection of Somatic Point Mutations in Impure and Heterogeneous Cancer Samples. Nat Biotechnol (2013) 31:213–9. doi: 10.1038/nbt.2514 PMC3833702 23396013

[4] Ramos AH Lichtenstein L Gupta M Lawrence MS Pugh TJ Saksena G. Oncotator: Cancer Variant Annotation Tool. Hum Mutat (2015) 36:E2423–9. doi: 10.1002/humu.22771 PMC7350419 25703262

[5] Wang K Li M Hakonarson H. ANNOVAR: Functional Annotation of Genetic Variants From High-Throughput Sequencing Data. Nucleic Acids Res (2010) 38:e164. doi: 10.1093/nar/gkq603 20601685 PMC2938201

[6] Kawaguchi S Higasa K Shimizu M Yamada R Matsuda F. HLA-HD: An Accurate HLA Typing Algorithm for Next-Generation Sequencing Data. Hum Mutat (2017) 38:788–97. doi: 10.1002/humu.23230 28419628

[7] Andreatta M Nielsen M. Gapped Sequence Alignment Using Artificial Neural Networks: Application to the MHC Class I System. Bioinformatics (2016) 32:511–7. doi: 10.1093/bioinformatics/btv639 PMC6402319 26515819

[8] Rosenthal R Larose Cadieux E Salgado R Al Bakir M Moore DA Hiley CT. Neoantigen-Directed Immune Escape in Lung Cancer Evolution. Nature (2019) 567:479–85. doi: 10.1038/s41586-019-1032-7 PMC6954100 30894752

[9] Gorris MAJ Halilovic A Rabold K van Duffelen A Wickramasinghe IN Verweij D. Eight-Color Multiplex Immunohistochemistry for Simultaneous Detection of Multiple Immune Checkpoint Molecules Within the Tumor Microenvironment. J Immunol (2018) 200:347–54. doi: 10.4049/jimmunol.1701262 29141863

[10] Pang X Qian J Jin H Zhang L Lin L Wang Y. Durable Benefit From Immunotherapy and Accompanied Lupus Erythematosus in Pancreatic Adenocarcinoma With DNA Repair Deficiency. J Immunother Cancer (2020) 8:e000463. doi: 10.1136/jitc-2019-000463 32636238 PMC7342819

[11] Li K Luo H Huang L Luo H Zhu X. Microsatellite Instability: A Review of What the Oncologist Should Know. Cancer Cell Int (2020) 20:16. doi: 10.1186/s12935-019-1091-8 31956294 PMC6958913

[12] Knijnenburg TA Wang L Zimmermann MT Chambwe N Gao GF Cherniack AD. Genomic and Molecular Landscape of DNA Damage Repair Deficiency Across The Cancer Genome Atlas. Cell Rep (2018) 23:239–54. doi: 10.1016/j.celrep.2018.03.076 PMC5961503 29617664

[13] Sanmamed MF Chen L. A Paradigm Shift in Cancer Immunotherapy: From Enhancement to Normalization. Cell (2018) 175:313–26. doi: 10.1016/j.cell.2018.09.035 PMC6538253 30290139

[14] Barbetta A Hsu M Tan KS Stefanova D Herman K Adusumilli PS. Definitive Chemoradiotherapy *Versus* Neoadjuvant Chemoradiotherapy Followed by Surgery for Stage II to III Esophageal Squamous Cell Carcinoma. J Thorac Cardiovasc Surg (2018) 155:2710–21. doi: 10.1016/j.jtcvs.2018.01.086 PMC5960990 29548582

[15] Hunder NN Wallen H Cao J Hendricks DW Reilly JZ Rodmyre R. Treatment of Metastatic Melanoma With Autologous CD4+ T Cells Against NY-ESO-1. N Engl J Med (2008) 358:2698–703. doi: 10.1056/NEJMoa0800251 PMC3277288 18565862

[16] ASCO Abstract 9077 (2019). Available at: https://meetinglibrary.asco.org/record/174379/abstract.

[17] Yuen Teo M Seier K Ostrovnaya I Regazzi AM Kania BE Moran MM. Alterations in DNA Damage Response and Repair Genes as Potential Marker of Clinical Benefit From PD-1/PD-L1 Blockade in Advanced Urothelial Cancers. J Clin Oncol (2018) 36:1685–94. doi: 10.1200/JCO.2017.75.7740 PMC6366295 29489427

